# Leukocyte-Rich vs. Leukocyte-Poor Platelet-Rich Plasma for the Treatment of Knee Osteoarthritis

**DOI:** 10.3390/biomedicines11010141

**Published:** 2023-01-06

**Authors:** Ashim Gupta, Madhan Jeyaraman, Anish G. Potty

**Affiliations:** 1 Regenerative Orthopaedics, Noida 201301, UP, India; 2 Future Biologics, Lawrenceville, GA 30043, USA; 3 BioIntegrate, Lawrenceville, GA 30043, USA; 4 South Texas Orthopaedic Research Institute (STORI Inc.), Laredo, TX 78045, USA; 5 Indian Stem Cell Study Group (ISCSG) Association, Lucknow 226010, UP, India; 6 Department of Orthopaedics, Faculty of Medicine, Sri Lalithambigai Medical College and Hospital, Dr. MGR Educational and Research Institute, Chennai 600095, TN, India

Knee osteoarthritis (OA) is a well-established form of OA and accounts for nearly 4/5 of global OA burden [1]. The incidence of knee OA has continued to rise in recent decades and shows no signs of slowing down [1]. Its pathophysiology entails synovial tissue inflammation and articular cartilage worsening, resulting in unbearable pain and loss of function [2]. Presently, knee OA is managed through traditional treatment modalities, including pharmacological methods such as non-steroidal anti-inflammatory drugs (NSAIDs), opioids and corticosteroids; non-pharmacological methods such as weight loss, diet control, physical therapy and activity adjustment; and surgery such as knee arthroplasty (especially in advanced stages of knee OA), when conventional treatment modalities have been unsuccessful [2]. These conventional options have contraindications and side-effects, and generally aim to minimize pain as opposed to targeting the fundamental pathology [2].

The last decade has seen a considerable increase in the use of biologics, including autologous biologics such as platelet-rich plasma (PRP), in regenerative medicine applications [3]. Several studies, including randomized controlled trials (RCTs), systematic reviews and meta-analyses, have demonstrated the superior efficacy of PRP compared to saline, corticosteroids and hyaluronic acid for the treatment of knee OA [4,5]. In addition, preclinical studies have demonstrated the disease-modifying abilities of PRP, including the reduction of synovial inflammation and the mitigation of cartilage deterioration [6]. Nevertheless, in spite of the enormous therapeutic potential of PRP, several questions remain. Specifically, different methods of PRP preparation exist that yield formulations with varying compositions and characteristics. Moreover, limited information is available regarding the most appropriate PRP formulations for certain clinical indications [7]. Herein, we focus on the presence of leukocytes, a highly discussed attribute related to the efficacy of PRP and a key discriminant in differentiating PRP formulations. Some studies have raised concerns regarding the pro-inflammatory nature of leukocytes present in PRP formulations [8]. Some in vitro studies have shown that high leukocyte content in the PRP can upsurge the expression of catabolic cascades, along with expression of inflammatory cytokines including IL-1 and TNF-α [9,10,11]. Similar findings have been reported in preclinical studies [12,13]. Based on these findings, some studies have recommended the utilization of leukocyte-poor PRP (LP-PRP) for the treatment of knee OA. On the other hand, despite the increased pro-inflammatory cytokines in leukocyte rich PRP (LR-PRP), some in vitro studies have reported that the utilization of LR-PRP could be beneficial in the treatment of knee OA due to the interaction between platelets and neutrophils. Platelets and neutrophils can hinder the conversion of leukotrienes into lipoxin, thereby prompting the resolution phase of the healing cascade [14,15]. Additionally, a study by Dohan Ehrenfest et al., reported the production of large amounts of VEGF from platelets by neutrophils [16]. Furthermore, Assirelli et al., reported a five-fold increase in the secretion of anti-inflammatory cytokines including IL-4 and IL-10 [17]. Despite knowledge of the concurrent anabolic and catabolic effects of leukocytes, there are limited high-level clinical trials available to help researchers understand the true clinical effects of leukocytes in PRP formulations utilized to treat knee OA. In this editorial, we focus on a recently published prospective clinical study titled “Leukocyte-rich versus Leukocyte-poor Platelet-Rich Plasma for the Treatment of Knee Osteoarthritis: A Double-Blind Randomized Trial”, in which the safety and efficacy of LR-PRP and LP-PRP were compared [18].

In this study by Di Martino et al. (ClinicalTrials.gov identifier: NCT02923700) [18], a total of 192 patients were enrolled in line with inclusion and exclusion criteria, which were as follows. Inclusion criteria: male and female patients affected by unilateral symptomatic knee OA with a history of chronic pain or swelling (at least 4 months); aged between 18 and 80 years; imaging findings of knee OA (Kellgren–Lawrence grade 1–3); and failed results after at least 2 months of non-operative treatment). Exclusion: aged > 80 years; Kellgren–Lawrence grade 4; bilateral knee OA; history of trauma or intra-articular injection therapy within 6 months before treatment, or knee surgery within 12 months; major axial deviation (varus > 5°, valgus > 5°); the presence of any concomitant knee lesion causing pain or swelling (e.g., untreated knee instability, meniscal pathologies, a focal chondral or osteochondral lesion requiring surgery); neoplasms; systemic disorders (e.g., uncontrolled diabetes); metabolic disorders of the thyroid; severe cardiovascular diseases; rheumatoid arthritis; inflammatory arthropathy; hematological diseases; infections; immunodepression; anticoagulant or antiaggregant therapy; the use of nonsteroidal anti-inflammatory drugs (NSAIDs) in the 5 days before blood harvest; and hemoglobin levels < 11 g/dL or a platelet count < 150,000/mm^3^ at blood harvest. Patients were randomly allocated 3-weekly intra-articular injections of either LR-PRP or LP-PRP. Both PRP formulations were prepared via a manual technique resulting in 5 mL aliquots of LR-PRP and LP-PRP, with a mean platelet concentration of 1146.8 × 10^9^/L and 1074.9 × 10^9^/L and a mean leukocyte concentration of 7991.4 × 10^6^/L and 0.1 × 10^6^/L, respectively. PRP was activated with 1 mL calcium gluconate prior to injection. All patients were assessed at baseline, and again at 2, 6 and 12 months after the last injection, for complications and adverse events; for the primary clinical outcome measure (the International Knee Documentation Committee (IKDC) subjective score); and for the secondary outcome measures (the Knee Injury and Osteoarthritis Outcome Score (KOOS) subscales, the EuroQol–visual analog scale (EQ-VAS) and the Tegner scale). Of the 192 patients, 11 patients were excluded due to personal reasons and 6 were excluded due to unavailability for follow-up visits; this resulted in 90 patients in the LR-PRP group and 85 in the LP-PRP group. No severe adverse events were reported in either group, though 15 mild adverse events (knee pain, and joint warmth or swelling) were observed that resolved within few days with cold compression and rest. The rate of mild adverse effects was 12.2% for LR-PRP vs. 4.7% for LP-PRP, although the difference was not statistically significant. Statistically significant (*p* < 0.05) improvements were observed in all clinical outcome measures at the 12-month follow-up visit compared to the baseline for both LR-PRP and LP-PRP; however, no differences were observed between the two groups in terms of absolute values and improvements in other clinical scores. In addition, no significant difference in terms of failure was found between the two groups. This study was not without limitations, as also discussed by the study’s authors. Primarily, the study lacked a control group to assess the placebo effect, molecular analysis to ensure no effect of the leukodepletion filter on the composition of the PRP formulations, and imaging evaluations during follow-up visits. Despite these limitations, we applaud the efforts of the study authors, who showed that 3-weekly intra-articular injections of LR-PRP or LP-PRP led to similar clinical improvement at 12-month follow-up in patients suffering with symptomatic knee OA.

Interestingly, the results from this study are in accordance with a recently published systematic review and meta-analysis by Kim et al. [19], who demonstrated that intra-articular injection of either LR-PRP or LP-PRP resulted in improvements above the minimal clinically important difference, in terms of both pain and function, in patients suffering with knee OA 12 months post-injection. However, in addition to including RCTs, this study also included prospective comparative studies and case series, leading to inherent heterogeneity attributed to uncontrolled bias. In fact, only one RCT was included. In addition, heterogeneity in injection volume and frequency was not considered. Despite these limitations, the findings of this systematic review and meta-analysis supported the potential use of either LR-PRP or LP-PRP for the treatment of knee OA.

In conclusion, despite their constraints, these studies shed light on this contentious facet of PRP and justify the need for high-powered, multi-centered, double-blinded RCTs with a longer follow-up duration to further compare the efficacy of LR-PRP with LP-PRP, and to further optimize PRP treatment for patients suffering with knee OA. As of 29 December 2022, only one ongoing clinical trial is registered on clinicaltrials.gov (search terms: “knee osteoarthritis” and “Platelet-rich Plasma” or “leukocyte-rich Platelet-rich Plasma” or “leukocyte-poor Platelet-rich Plasma”) that directly compares the efficacy of LR-PRP with LP-PRP. This trial is summarized in Table 1.

## Figures and Tables

**Table 1:** Ongoing clinical trial registered on ClinicalTrials.gov from 29 December 2022 comparing leukocyte-rich vs. leukocyte-poor Platelet-rich Plasma for treatment of knee osteoarthritis.

Study Identifier	Biologic (Description)	Study Phase; Estimated Enrollment (N)	Primary Outcome Measure(s)	Recruitment Status	Country
NCT04187183	Fresh PRP with concentrated leukocytes vs. Fresh PRP without concentrated leukocytes	Not applicable; N = 132	IKDC-subjective score (International Knee Documentation Committee) (Time Frame: 12 months). As a specific subjective rating scale for the knee, this scoring system is one of the most reliable tools for assessing knee diseases. The survey examines three categories: symptoms, sport activity and knee function.	Recruiting	Italy
